# Supplementary figures and images for: Conformational dynamics and putative substrate extrusion pathways of the N-glycosylated outer membrane factor CmeC from Campylobacter jejuni
Source: PLoS Comput Biol. 2023 Jan 13;19(1):e1010841. doi: 10.1371/journal.pcbi.1010841 (PMC9879487; doi:10.1371/journal.pcbi.1010841)

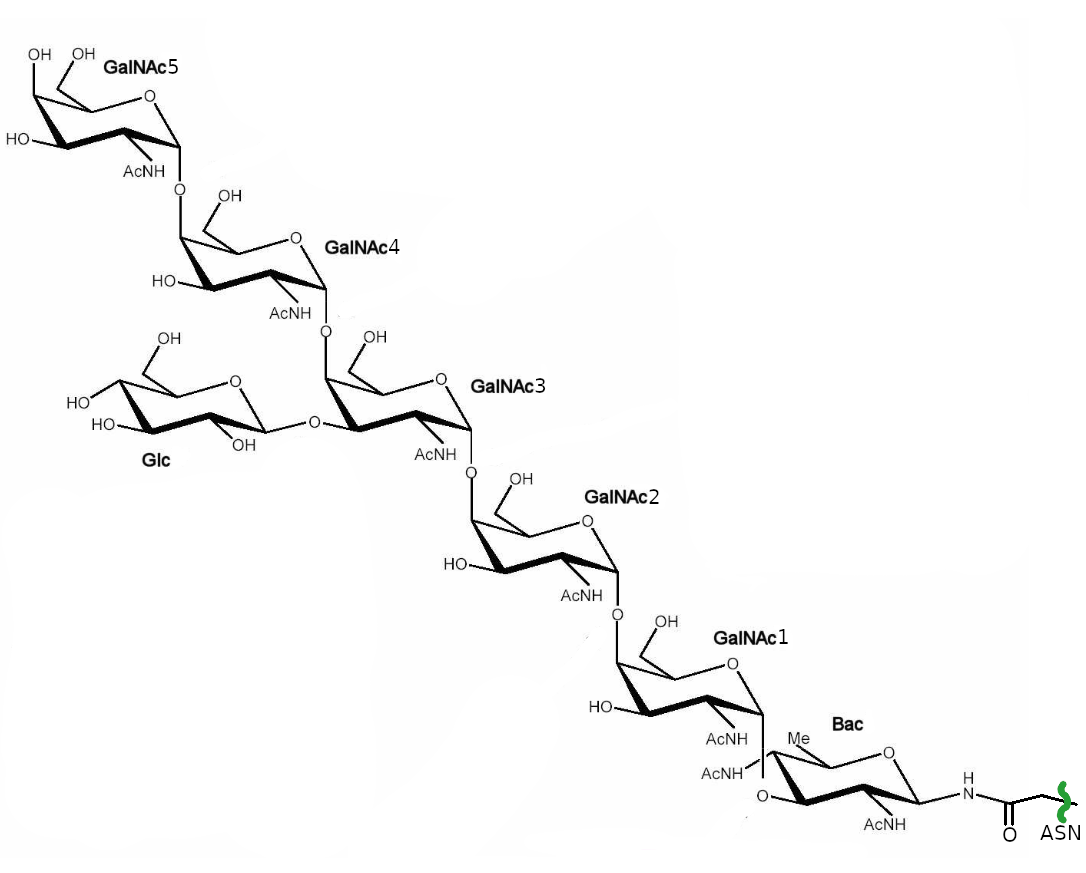

Supplement: S1 Fig — (TIF) [file pcbi.1010841.s001.tif]

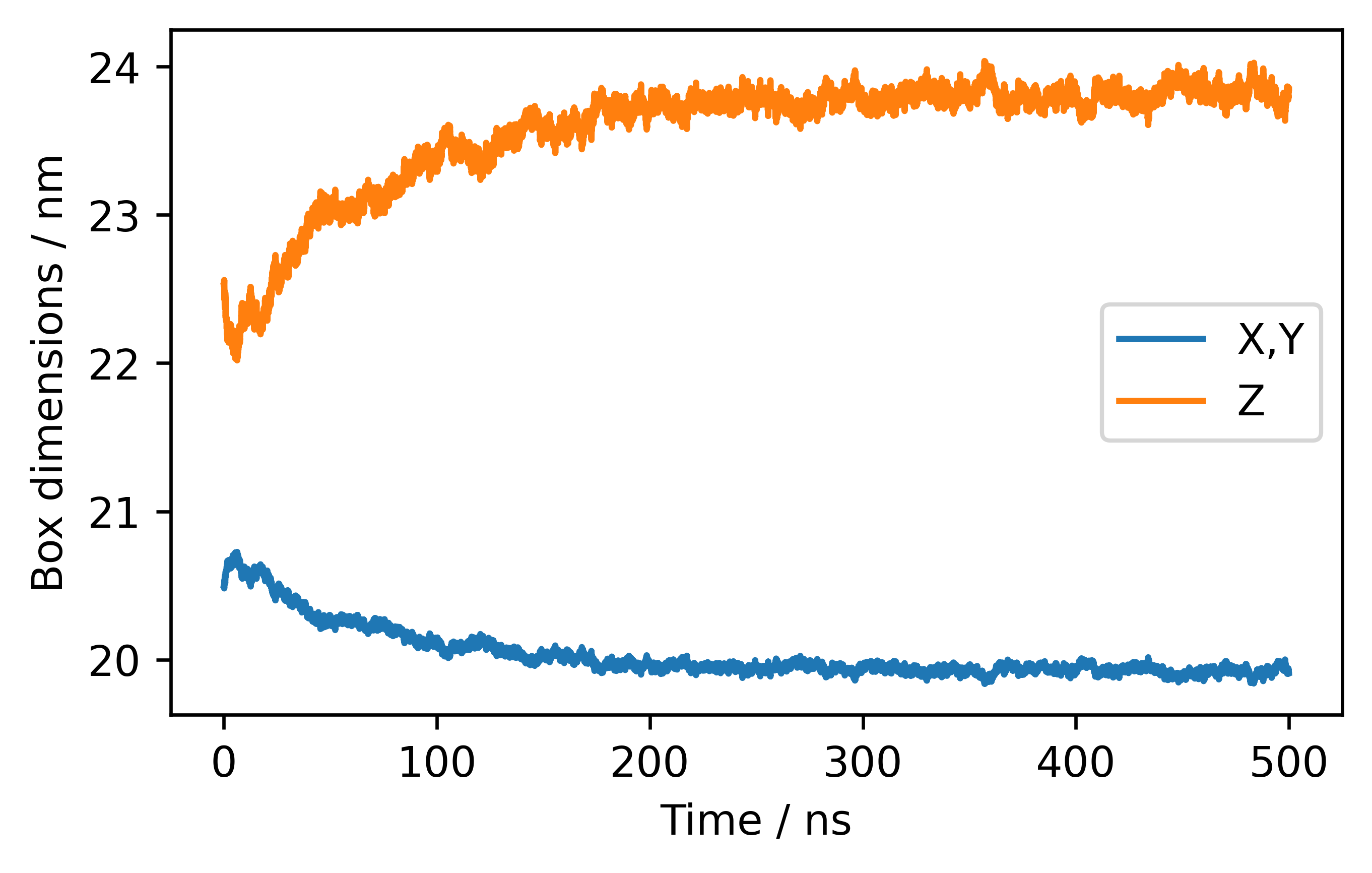

Supplement: S2 Fig — X and Y dimensions of the box are coupled (semi-isotropic pressure coupling used). Box dimensions are stable after ~300 ns. (TIF) [file pcbi.1010841.s002.tif]

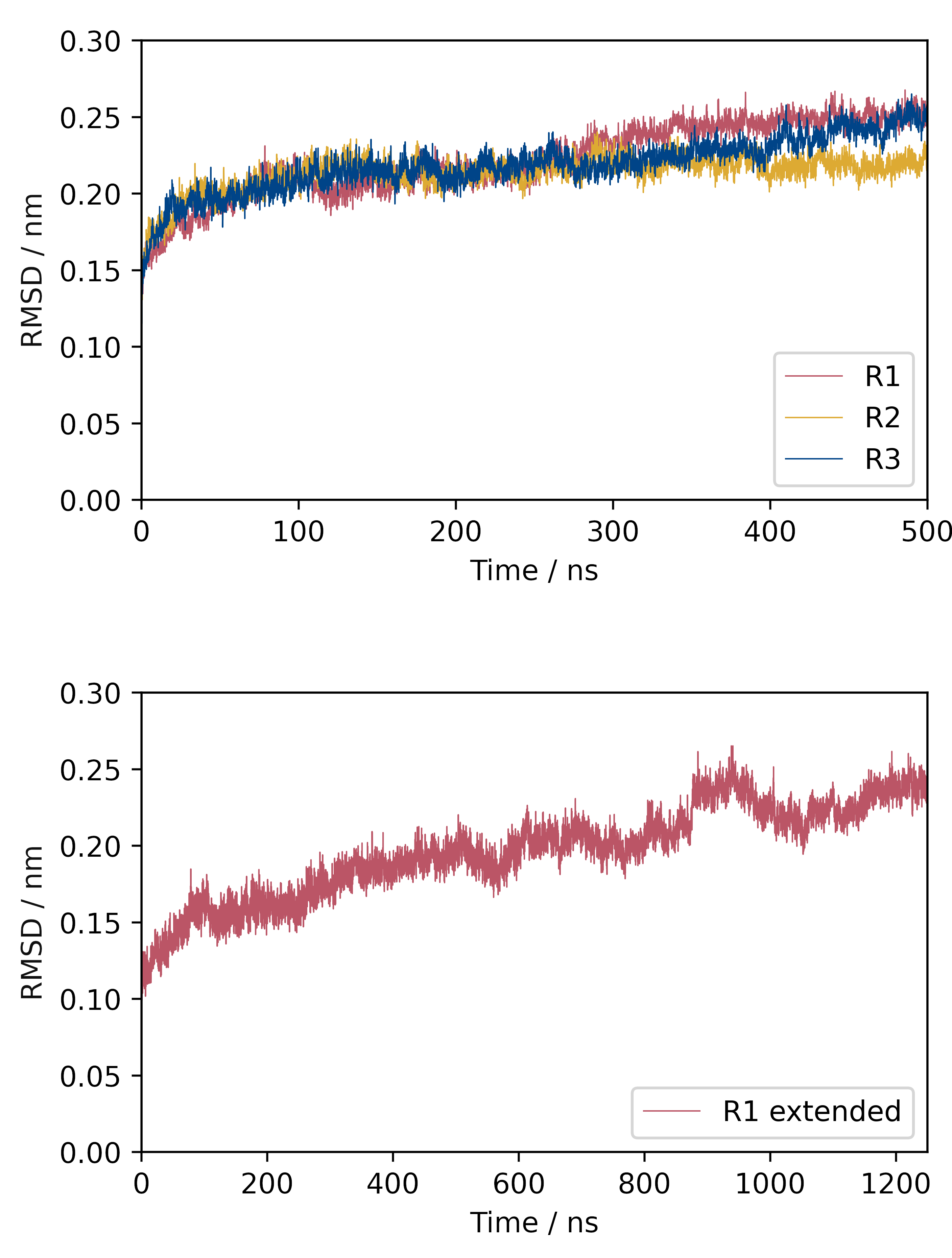

Supplement: S3 Fig — In all three replicates the RMSD is still increasing at 500 ns, indicating that CmeC is not yet fully equilibrated. Bottom: When one replicate was extended to 1250 ns, the backbone RMSD continued to increase. (TIF) [file pcbi.1010841.s003.tif]

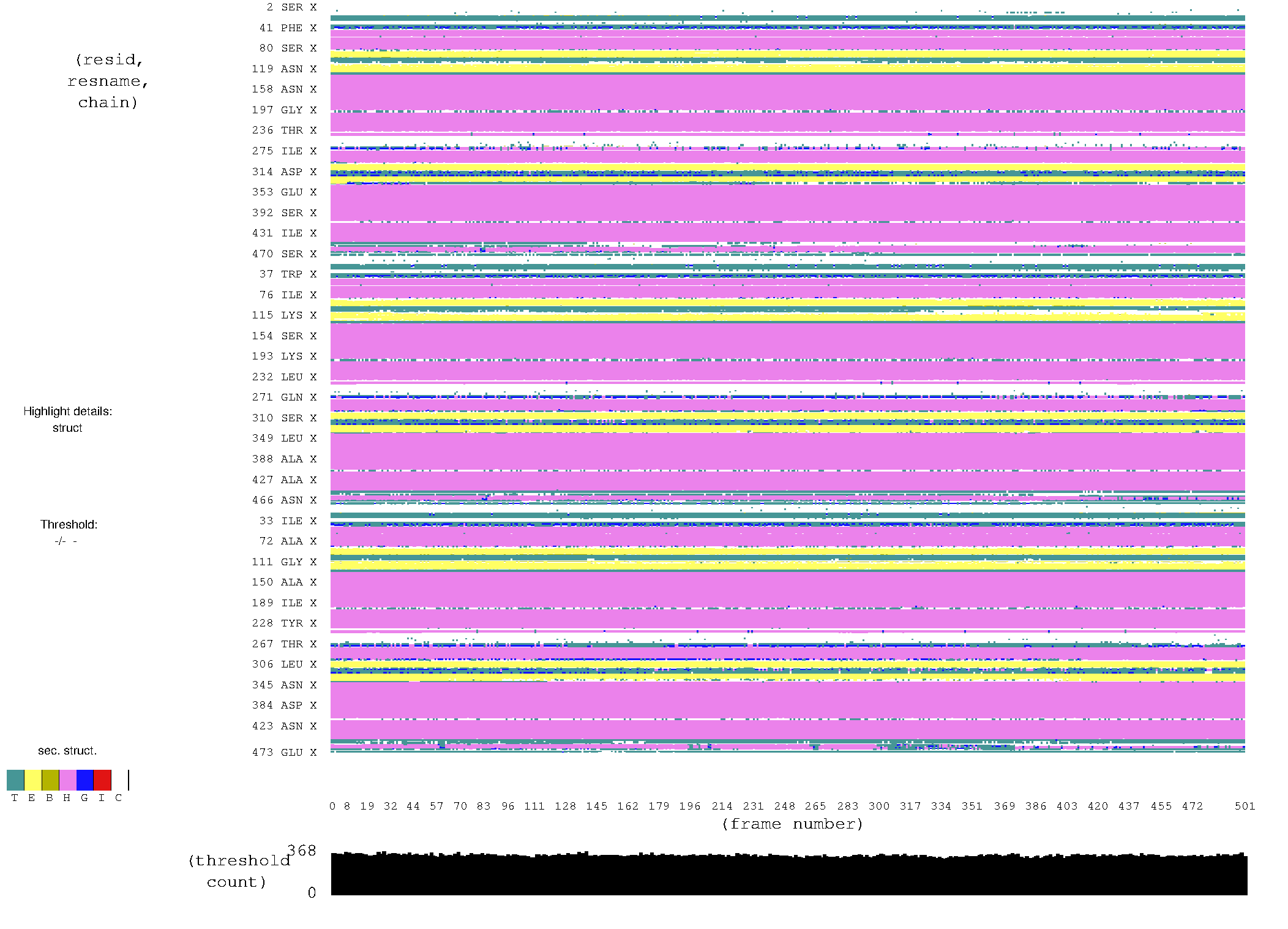

Supplement: S4 Fig — (TIF) [file pcbi.1010841.s004.tif]

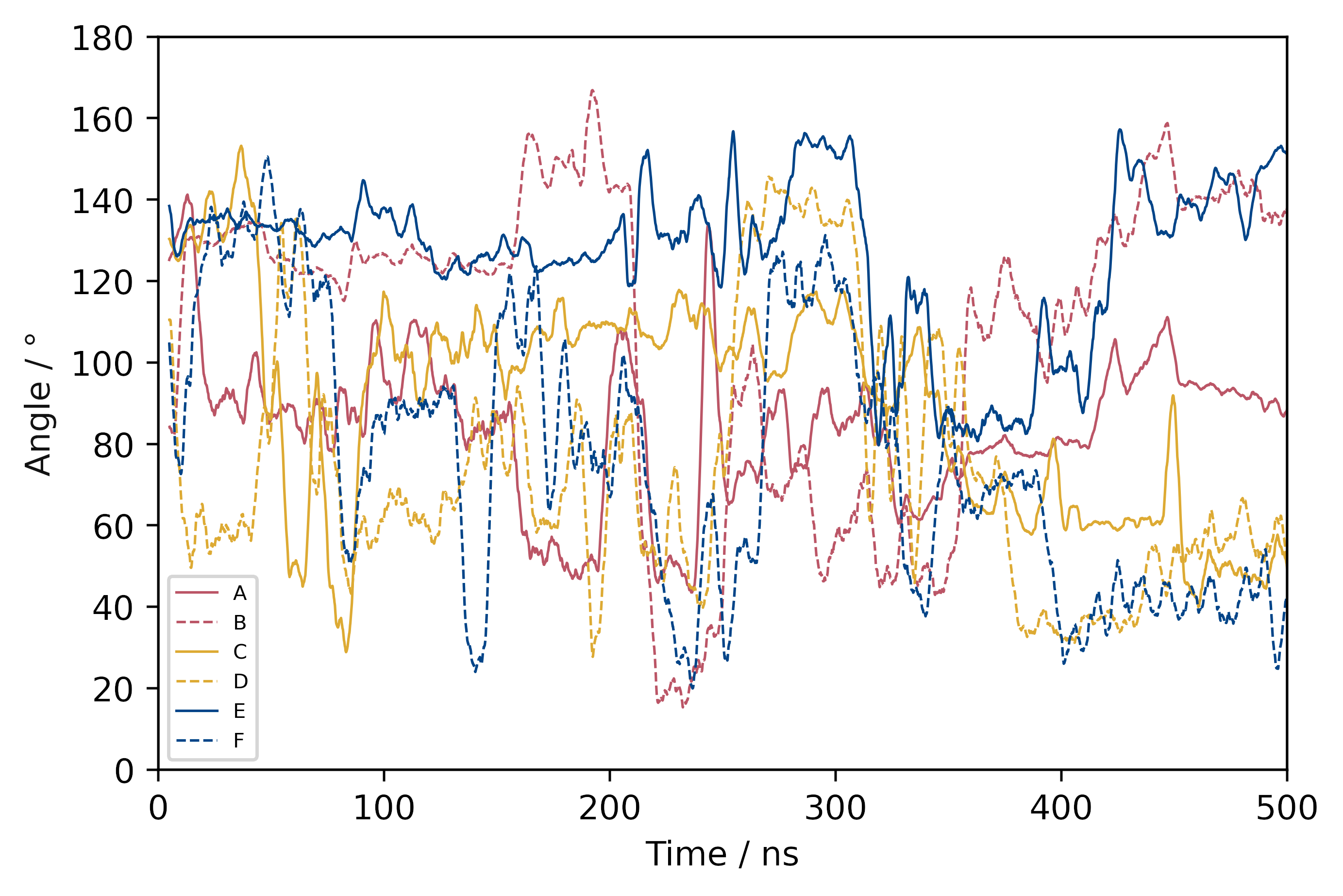

Supplement: S5 Fig — Timeseries data of the angle defined between the protein principal axis (approximately parallel to the z-axis) and the glycan vector (defined in Fig 4E). A wide variety of relative orientations are observed. (TIF) [file pcbi.1010841.s005.tif]

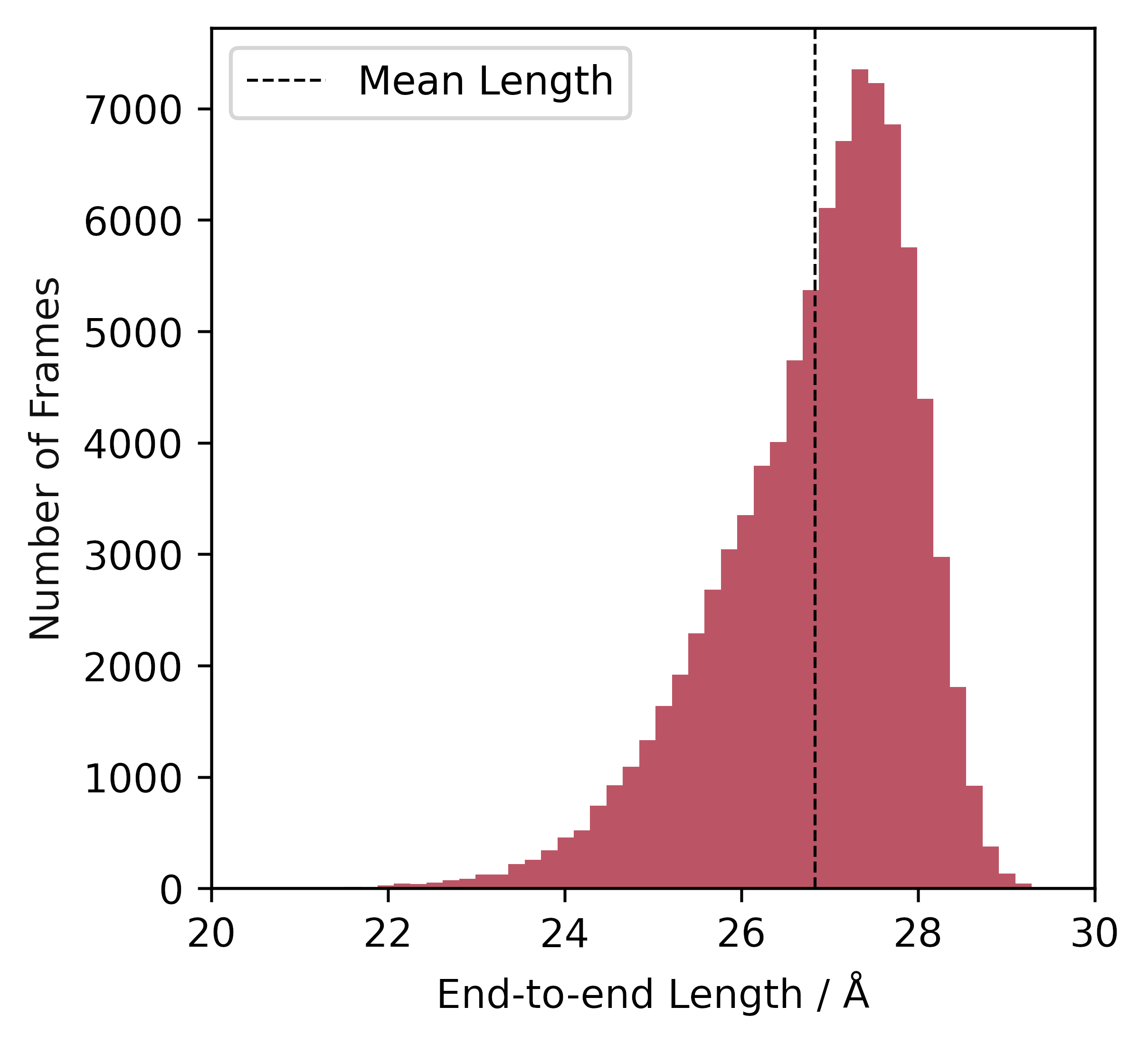

Supplement: S6 Fig — Ther is considerably more variation in this value than would be expected from a ‘rigid-rod’. (TIF) [file pcbi.1010841.s006.tif]

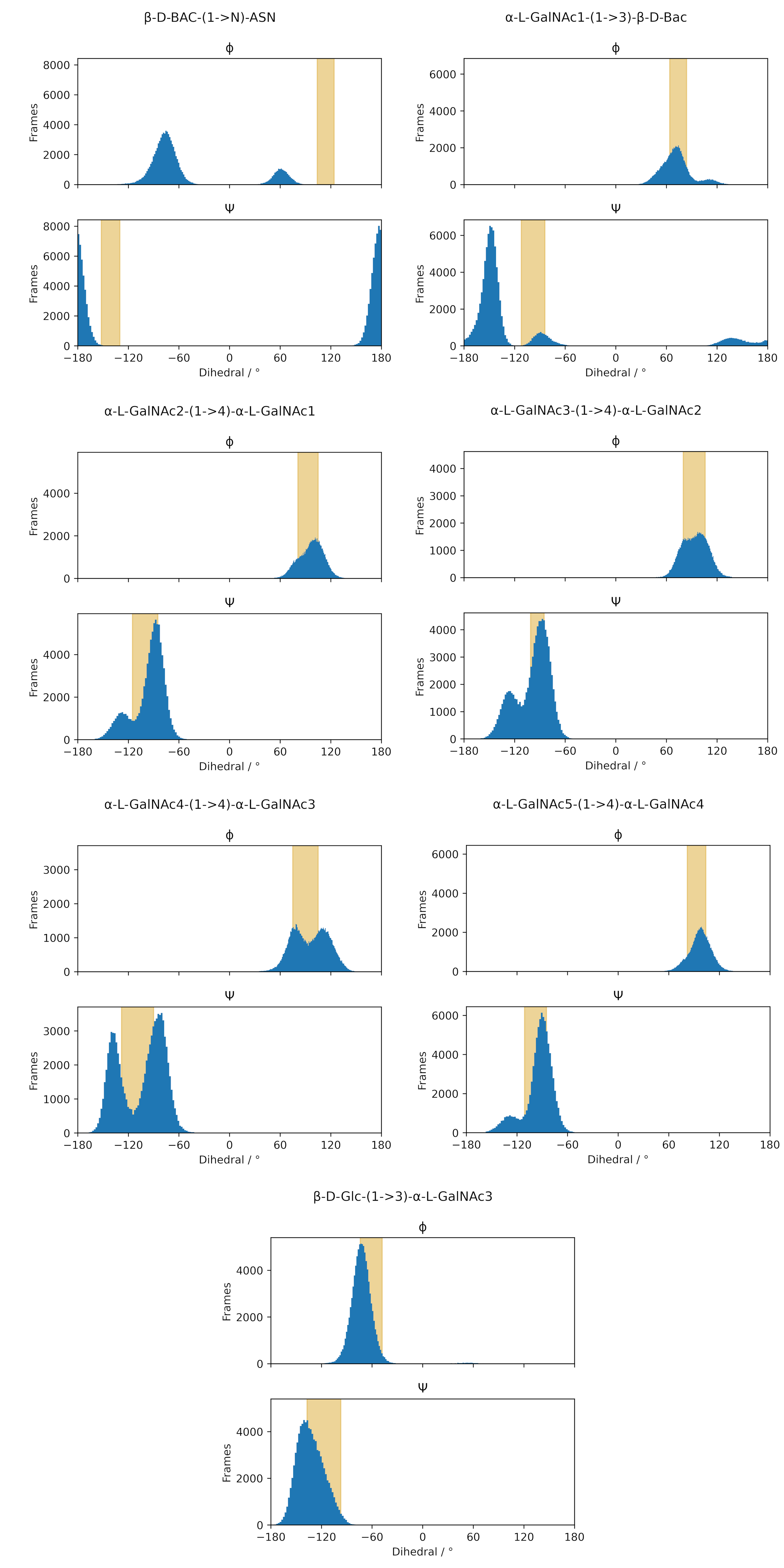

Supplement: S7 Fig — Data from our equilibrium simulations plotted in blue. Values defined by the mean ± standard deviation in Ref 60 for each linkage shaded in yellow (TIF) [file pcbi.1010841.s007.tif]

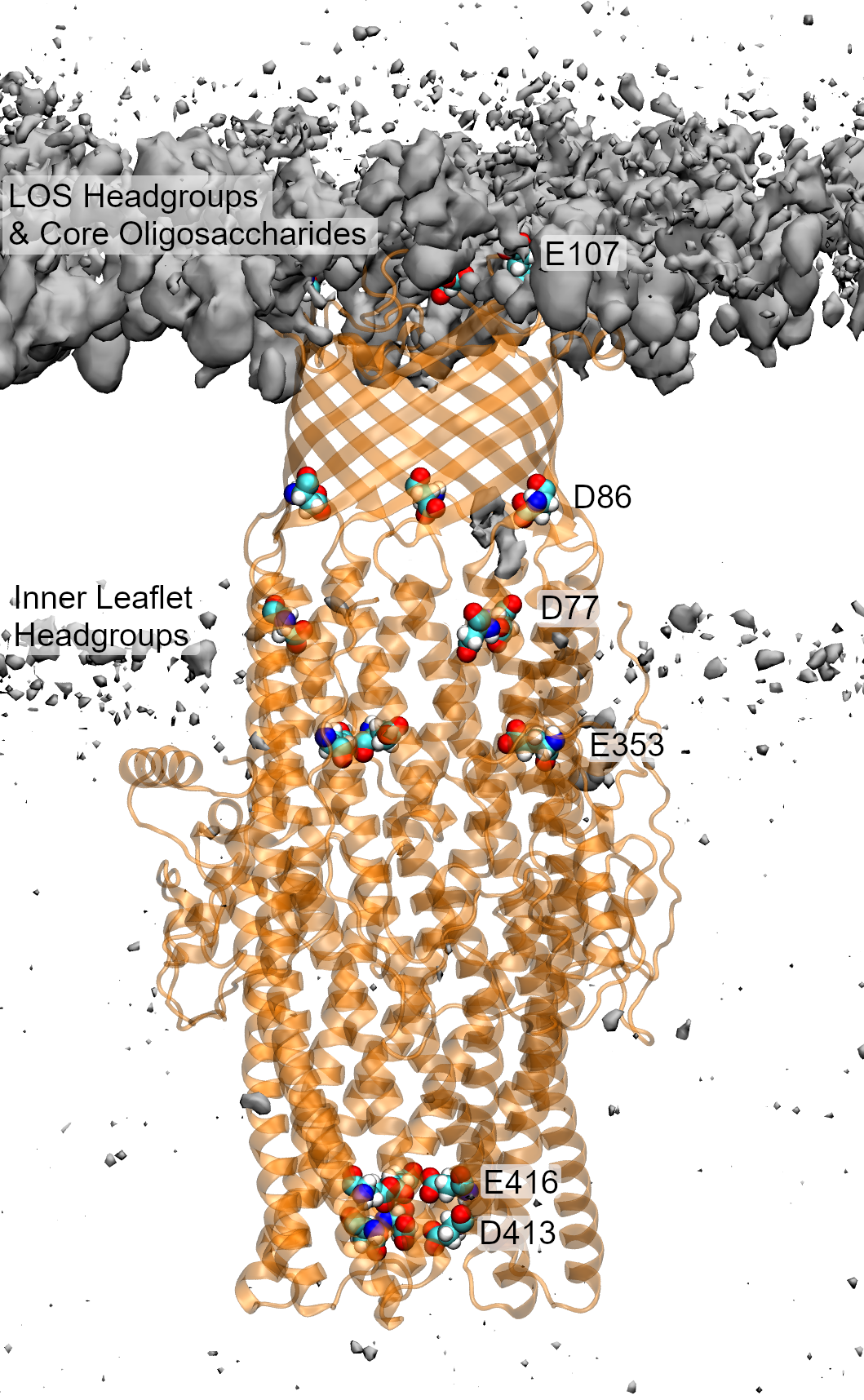

Supplement: S8 Fig — A small amount of potassium ion density is located near D86 within the channel. (TIF) [file pcbi.1010841.s008.tif]

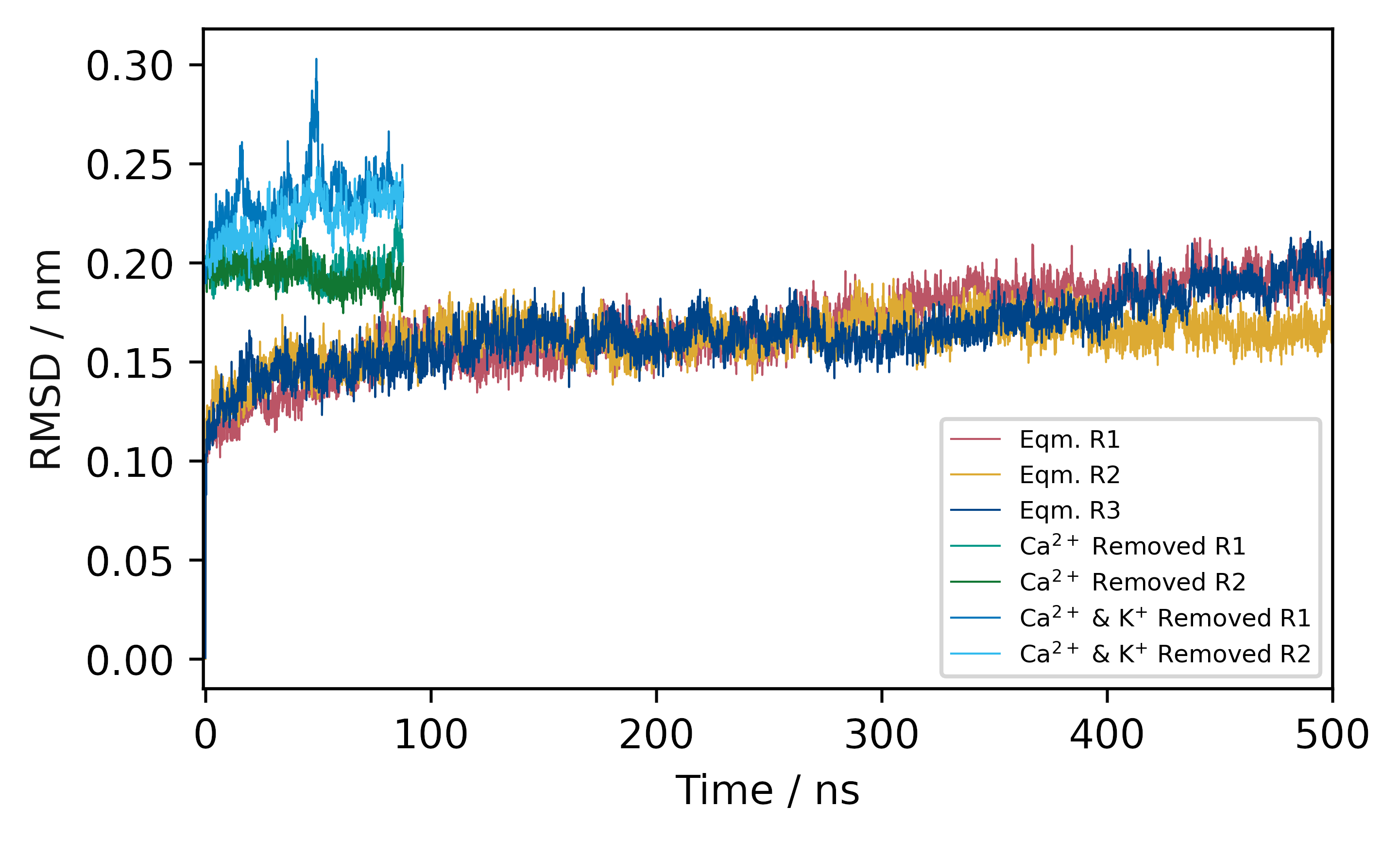

Supplement: S9 Fig — Systems where cations were removed saw increased RMSD values compared to equilibrium simulations, though this is most pronounced in systems where both calcium and potassium ions were removed. (TIF) [file pcbi.1010841.s009.tif]

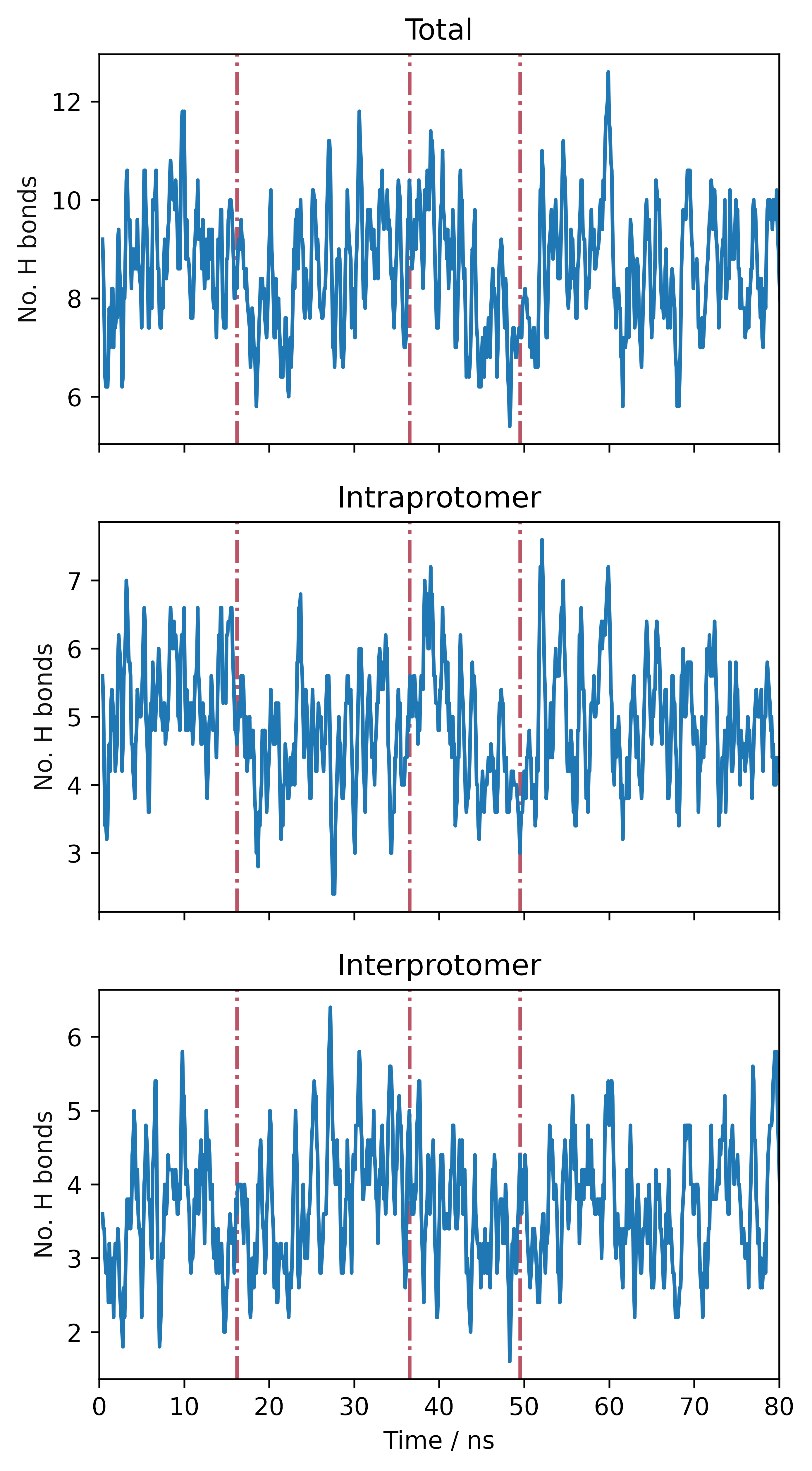

Supplement: S10 Fig — Dashed pink vertical lines indicate times at which there was a peak in TCAE416; there is no clear increase or decrease in the number of these hydrogen bonds (inter- or intraprotomer) correlated with peaks in TCA. (TIF) [file pcbi.1010841.s010.tif]

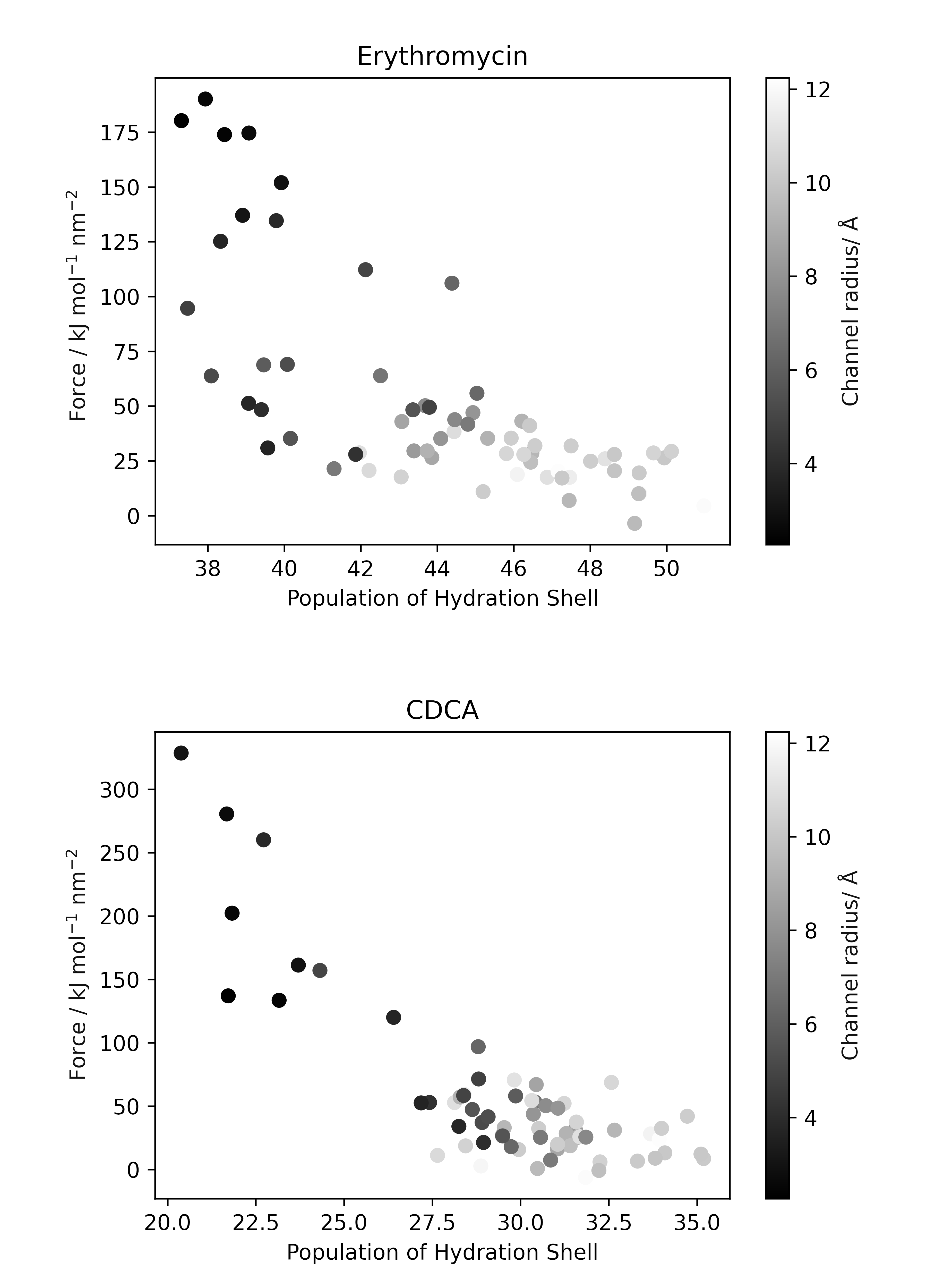

Supplement: S11 Fig — (TIF) [file pcbi.1010841.s011.tif]

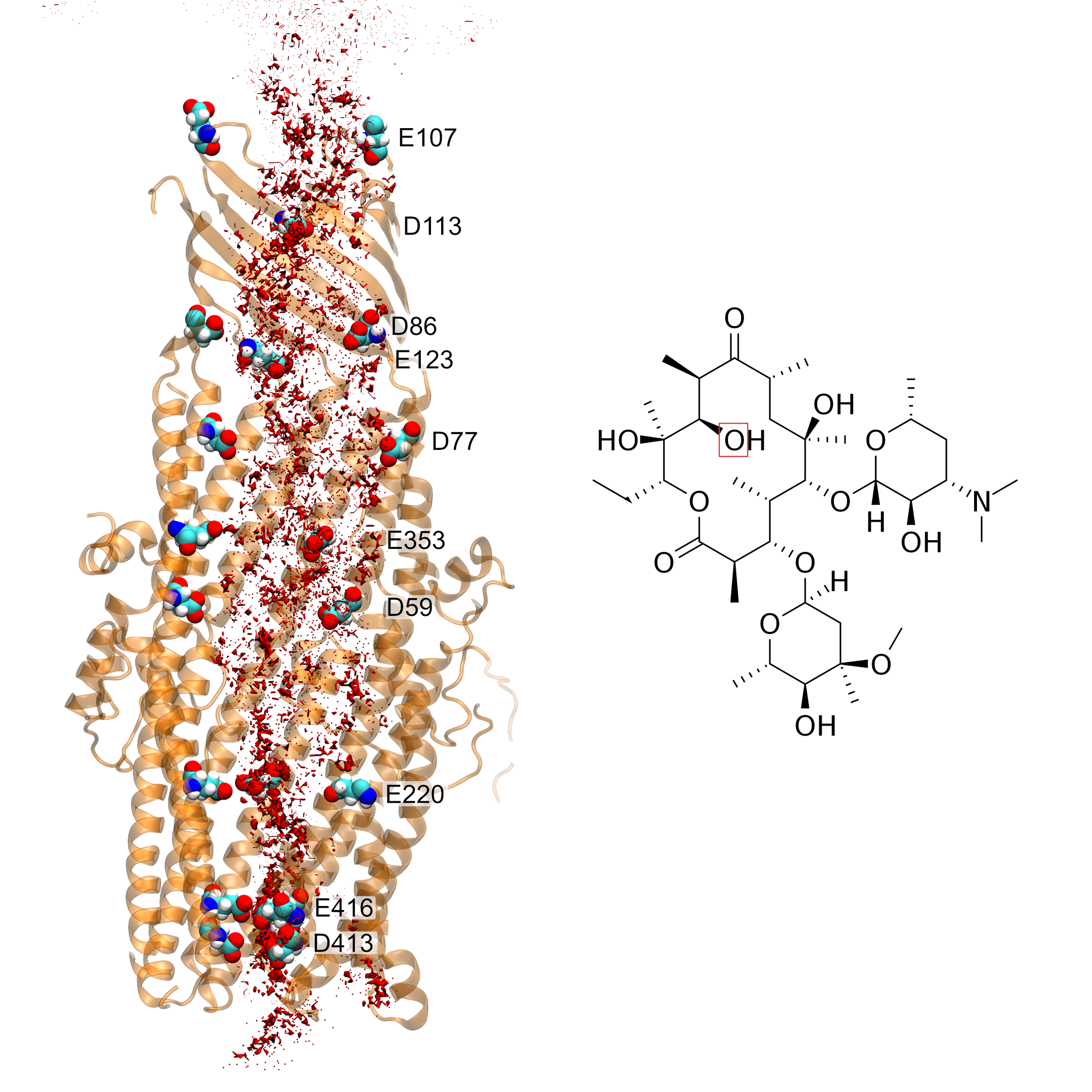

Supplement: S12 Fig — The occupancy is delocalised in the channel. The only clear localised region of high occupancy is at the periplasmic entrance, where the rotational freedom of erythromycin is limited due to the narrow channel diameter. (TIF) [file pcbi.1010841.s012.tif]

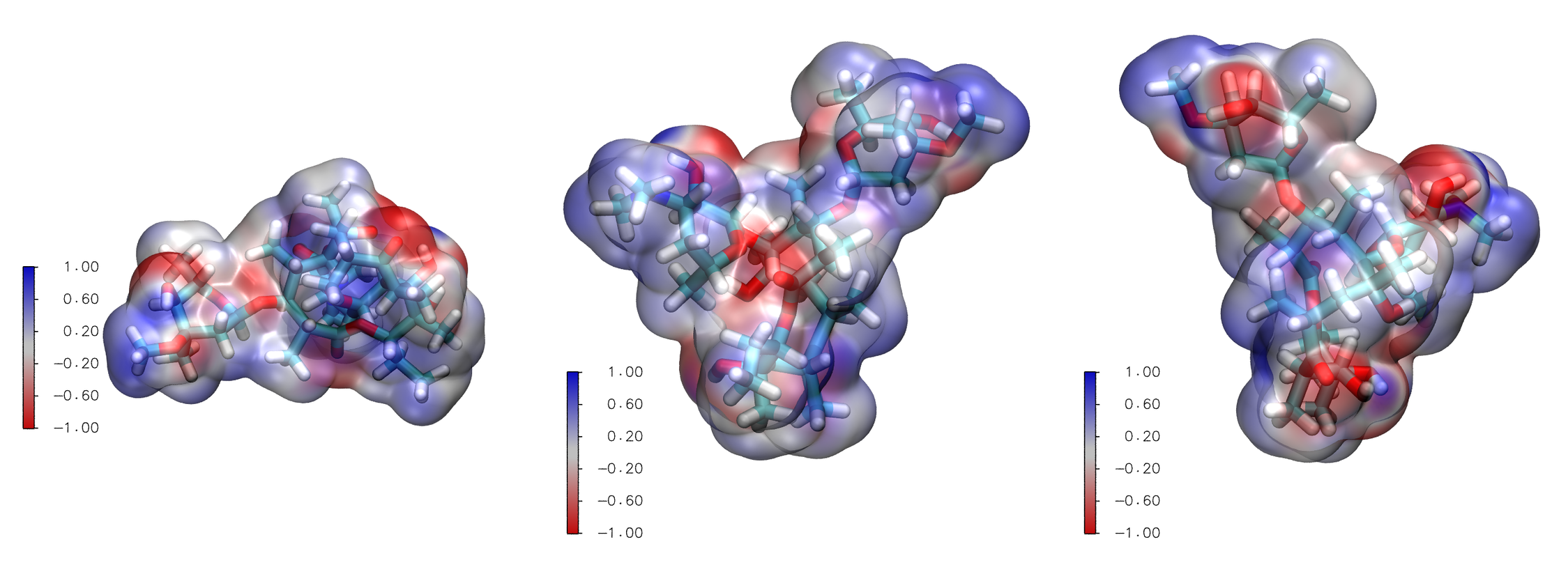

Supplement: S13 Fig — Colour scale: -1 V in red to +1 V in blue. Electronegative groups are distributed around the erythromycin molecule. (TIF) [file pcbi.1010841.s013.tif]

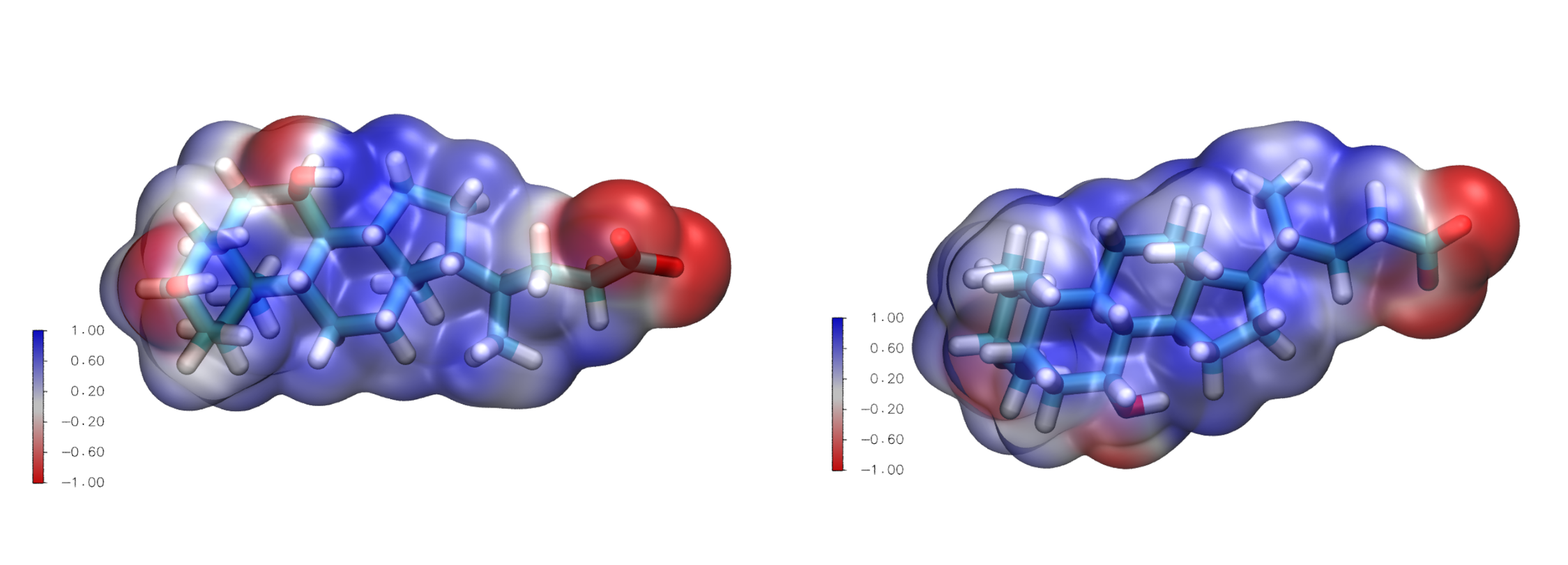

Supplement: S14 Fig — Colour scale: -1 V in red to +1 V in blue. While there are three electronegative functional groups in CDCA, the carboxylate group dominates. (TIF) [file pcbi.1010841.s014.tif]

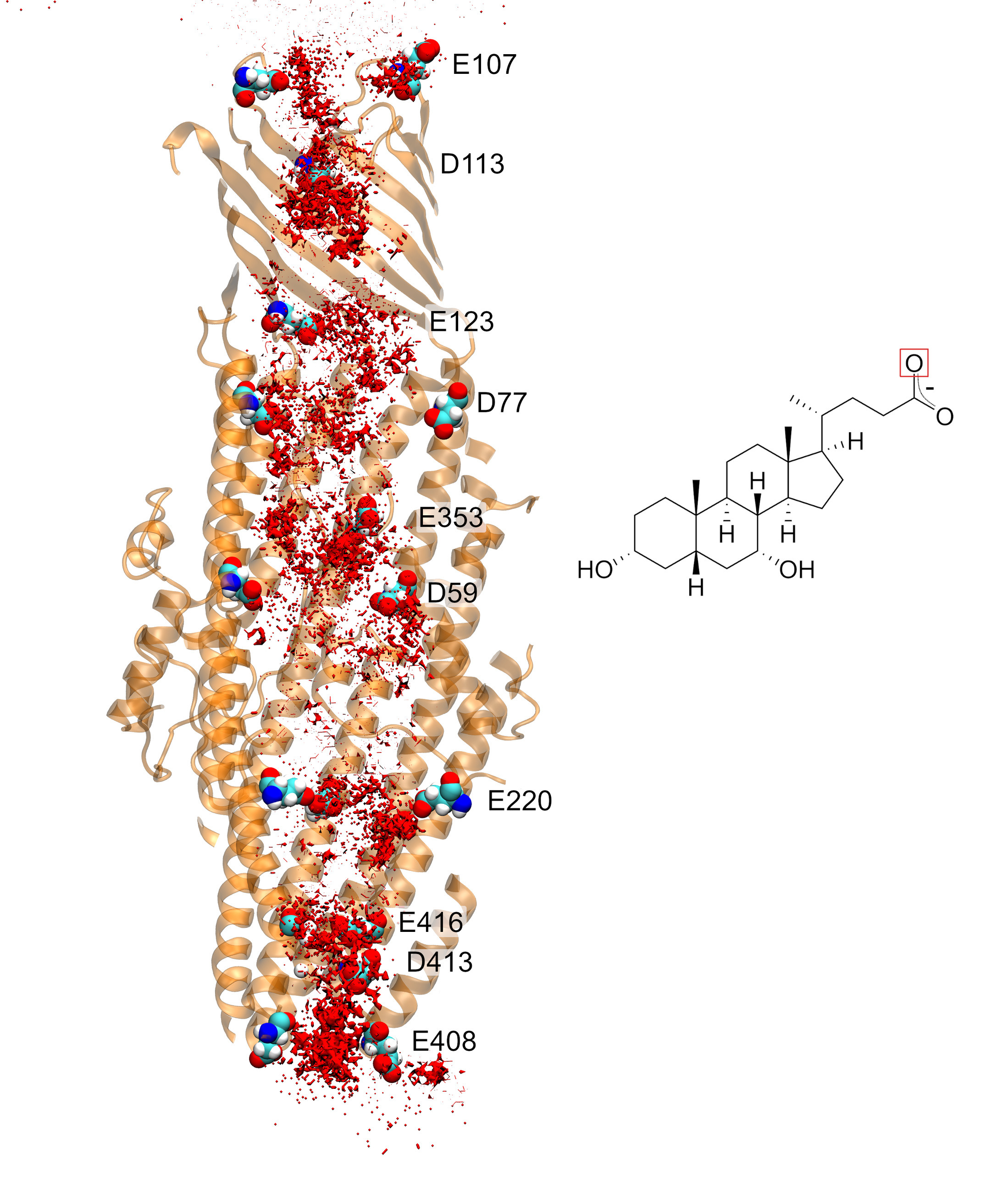

Supplement: S15 Fig — There are several localised regions of high within the channel. These align with acidic lining residues where CDCA is coordinating a bound protein-bound calcium ion. (TIF) [file pcbi.1010841.s015.tif]
