# Supplementary material for: Conformational dynamics and putative substrate extrusion pathways of the N-glycosylated outer membrane factor CmeC from Campylobacter jejuni
Source: PLoS Comput Biol. 2023 Jan 13;19(1):e1010841. doi: 10.1371/journal.pcbi.1010841 (PMC9879487; doi:10.1371/journal.pcbi.1010841)
Supplement: S1 Table — (PDF) [file pcbi.1010841.s016.pdf]

Supplementary Table 1: percentage variance explained by the first 10 eigenvectors identified via principal component analysis.

PC	% variance explained	Cumulative explained variance / %
1	17.0	17.0
2	8.4	25.4
3	6.9	32.3
4	3.8	36.0
5	3.3	39.4
6	3.2	42.5
7	2.3	44.8
8	2.0	46.8
9	1.9	48.8
10	1.7	50.4
