# Supplementary material for: Conformational dynamics and putative substrate extrusion pathways of the N-glycosylated outer membrane factor CmeC from Campylobacter jejuni
Source: PLoS Comput Biol. 2023 Jan 13;19(1):e1010841. doi: 10.1371/journal.pcbi.1010841 (PMC9879487; doi:10.1371/journal.pcbi.1010841)
Supplement: S2 Table — (PDF) [file pcbi.1010841.s017.pdf]

Supplementary Table 2: Mean values of Ψ and Φ for each linkage in the heptasaccharide, with associated standard deviation.

	Ψ / °	Φ / °
Bac-Asn	-55 ± 55	117 ± 9
GalNAc1-Bac	71 ± 18	-150 ± 31
GalNAc2-GalNAc1	97 ± 15	-96 ± 19
GalNAc3-GalNAc2	94 ± 15	-99 ± 20
GalNAc4-GalNAc3	96 ± 21	-105 ± 27
GalNAc5-GalNAc4	97 ± 13	-94 ± 18
Glc-GalNAc3	-72 ± 16	-133 ± 16
