# Supplementary material for: Conformational dynamics and putative substrate extrusion pathways of the N-glycosylated outer membrane factor CmeC from Campylobacter jejuni
Source: PLoS Comput Biol. 2023 Jan 13;19(1):e1010841. doi: 10.1371/journal.pcbi.1010841 (PMC9879487; doi:10.1371/journal.pcbi.1010841)
Supplement: Attachment — Submitted filename: Response_To_Reviewers.docx [file pcbi.1010841.s018.docx]

Responses to Reviewers’ Comments

## Reviewer 1

*In this manuscript, the authors conducted extensive molecular dynamics simulations to investigate the conformational dynamics of a N-glycosylated outer membrane factor CmeC and the possible extrusion pathway of small ligands which can be exported from the intracellular side to the extracellular side of the bilayer by this protein. The OMF is responsible for antibiotic resistance of the bacteria, Campylobacter jejuni, and therefore studying its mechanism of exporting small ligands is of interest to the community. The authors described in detail the conformation of the protein in equilibrium simulations, as well as how the saccharides affected its conformation. The authors also employed non-equilibrium simulations to pull two ligands across the channel. The authors concluded the manuscript with possible gates of the channel and a probable mechanism for negatively charged ligands using cations to climb a channel whose inner surface is full of acid residues. Overall, the technique used in this work is solid, the results were analyzed properly and the manuscript was written in reasonable quality. I only have some minor issues which the authors may want to consider before the publication of this manuscript:*

***The authors used both potassium ions and calcium ions in their system. However, in their work, they mentioned that they found little potassium density inside the channel, but notable calcium density. What would be the possible reason for this difference? Is this a possible artefact of the used force field (as far as I know, the current ff with fixed charges are not perfect to describe ions, particularly multivalent cations), or there was some more fundamental reason? This should be discussed in the manuscript.***

We thank the reviewer for bringing up this important point. While we do see both cations coordinating, commensurate with the different magnitudes of their charges they coordinate to different extents. We have now included an additional figure in the supplementary information (S8) to show the K^+^ coordination.

We also note that multivalent cation coordination has been observed experimentally [(1–3)](https://www.zotero.org/google-docs/?6rHiCR). From these experimental studies AND the simulations we report here coordination is particularly prominent at the periplasmic entrance, where there are two highly conserved rings of acidic residues. These experimental papers are now cited in the main text of the manuscript.

Limitations of the force field with respect to multivalent cations were also raised by Reviewer 3. It is possible that the coordination of calcium has been overestimated here; systematic overestimation of binding free energies between proteins and divalent cations has been documented [(4)](https://www.zotero.org/google-docs/?kVJ2pv). However, updates to the Charmm36 force field have addressed issues with the non-bonded interactions between cations and carboxylate moieties by increasing the Lennard-Jones parameter, σ [(5)](https://www.zotero.org/google-docs/?q4djxJ). Similar NBFIX force field corrections have been shown to drastically improve estimations of the cation-protein binding free energy [(6)](https://www.zotero.org/google-docs/?GLkCOX). We have used the CHARMM36m force field with these updated non-bonded parameters, and to the best of our knowledge this model is appropriate for the scope of this paper. We note that in a paper quantifying the overestimation of the binding free energy, the authors observed no calcium ion permeation events as the calcium was bound too tightly within the protein [(4)](https://www.zotero.org/google-docs/?rRk5Qz). This was not the case in our simulations: calcium ions were observed to bind and unbind within CmeC. To address the reviewers’ concerns, we have added an extra paragraph on our use of this model in the subsection titled ***“Cation binding sites within the channel”****.* Should we wish to calculate binding free energies or quantify conductance through the channel, a polarisable [(7,8)](https://www.zotero.org/google-docs/?uXfSbZ) or multi-point [(4)](https://www.zotero.org/google-docs/?fXUrkC) model would be used to ensure a reasonable accuracy.

***Also, the authors said that they used calcium ions to neutralize the negatively charged LOS lipids. Why is this particular cation necessary for this purpose? Please clarify.***

It has been shown experimentally that divalent cations are necessary for the integrity of the outer leaflet [(9–14)](https://www.zotero.org/google-docs/?kjVdNF). We and others have shown via simulations the role of divalent cations in maintaining the integrity of the outer membrane [(15–18)](https://www.zotero.org/google-docs/?45VV1M). This has also been shown in combined experimental and simulation studies we have done with Luke Clifton and Jeremy Lakey [(19)](https://www.zotero.org/google-docs/?EWGGyr) . We have clarified the importance of these cations in the text (second paragraph of the subsection titled ***“Cation binding sites within the channel”***) and added appropriate citations.

***On the one hand, the authors mentioned that they identified higher calcium density than the potassium density inside the channel, on the other hand, the authors said that removing the calcium only increased the TCA slightly, while removing the both types of ions increased the TCA significantly. The reason should be explained. Why minor potassium had more dramatic effects than the more enriched calcium?***

When both potassium and calcium ions are present, calcium preferentially occupies the coordination sites at the periplasmic constriction (the two rings of acidic residues, D413 and E416). This is due to the increased electrostatic attraction between calcium and the acidic residues when compared to potassium (Ca^2+^ versus K^+^), and may also be due to systematic overestimation of the binding of calcium in proteins [(4)](https://www.zotero.org/google-docs/?AlmU4o). This does not mean that potassium cannot occupy this site: when calcium is removed and not allowed to re-enter, potassium ions move into this site to be coordinated by the rings of aspartate and glutamate residues, thus the coiled-coil domain remains closed. When all cations are removed from the channel and none allowed to re-enter, the coiled-coil domain opens partially as there is no central cation to pull this end closed (and therefore repulsion between adjacent acidic residues which will encourage opening). Statements have been added to the 6th and 7th paragraphs of the subsection ***“Cation binding sites within the channel”*** to make this point clear in the text.

***Probably a comparison of the distribution of two different ions in the channel would be helpful: this distribution will show if the potassium is really not distributed in the channel or if they are just not coordinate stably with the acid residues.***

As mentioned above, a plot similar to Figure 7A was generated for the potassium ions, and is included in the supplementary information (new Supplementary Figure S7). We note that there is some potassium ion density within the channel, though not as much as the calcium ion density. The potassium ion density is mainly found near to D86. Given the additional discussion we have added about ions to the text, we feel we have now covered this important point.

***The authors performed principal component analyses in their work. The three simulation replicates showed complete different conformational space according to the principal components, what does this mean actually? It doesn’t seem to be a result of insufficient sampling.***

Each trajectory by itself is sampling only a subset of the subspace available to the protein, and all are sampling subspaces unique from each other, i.e. the motion of each replicate is different. By taking these trajectories in combination we get a greater coverage of the subspace. We have now made it clearer in the text that the combined data from the three simulations serves to extend the sampling that could be achieved by a single simulation, and have toned down the comments about insufficient sampling. We thank the reviewer for the reassuring comments about this issue.

***Also, how the PCA analyses were conducted is not clear. Did the authors conducted PCA for each trajectory separately, or they combined all of the trajectories and then conducted PCA for the combined frames all together? If the former, did the corresponding principal components from three trajectories indicate the same collective motions? Please clarify.***

To conduct the Principal Component Analysis, the three trajectories were concatenated. The PCA was applied to this combined trajectory. Each of the three replicate trajectories was then projected along the first principal components identified. This has been clarified in the text (second paragraph of the subsection titled ***“CmeC Stability within the Membrane”***)

***The authors said the conformational space of three trajectories are unique from the crystal structure, but the PCs of the crystal structure was not labeled in Fig. 2. Besides, how much percentage did the first three PCs account for among all of the PCs (in another word, was it enough to only look at the first three PCs)?***

The projection of the crystal structure onto the first three principal components corresponds to the origin, (0,0,0). A clarifying statement has been made in the caption of Figure 2.

The first three principal components explain a third of the variance. The variance explained by the first 10 principal components has now been included in the supplementary information (Supplementary Table 1). It is also included below:

| **PC** | **% variance explained** | **Cumulative explained variance / %** |
| --- | --- | --- |
| 1 | 17.0 | 17.0 |
| 2 | 8.4 | 25.4 |
| 3 | 6.9 | 32.3 |
| 4 | 3.8 | 36.0 |
| 5 | 3.3 | 39.4 |
| 6 | 3.2 | 42.5 |
| 7 | 2.3 | 44.8 |
| 8 | 2.0 | 46.8 |
| 9 | 1.9 | 48.8 |
| 10 | 1.7 | 50.4 |

We acknowledge that the first three principal components do not explain a substantial proportion of the variance. PCA was not sufficient to reduce the dimensionality particularly well as there were no clear major motions within the protein backbone. However, it is not possible to concisely visualise projections along more than three eigenvectors; to visualise that each replicate samples a unique subset of the subspace, we have proceeded with Figure 2B. We have added a statement in the text on the variance explained by the first three eigenvectors.

***The authors mentioned the two constriction regions at the periplasmic entrance and the extracellular exit in the introduction section, however, it is not clear where these regions are in Fig. 1. Of course, the positions were elaborated later in the manuscript, but I would suggest also label them in Fig. 1.***

Additional labels have been added to Figure 1B, and an additional statement on these constricting residues added to the caption of Figure 1.

***CDCA was supposed to utilize cations to climb the acid residue ladders of the channel, and this process was supposed to increase the ratio of the frames in which calcium ions were coordinated by acid residues. From Table 1, we can see that coordination of calcium by D77 was increased more significantly than the other residues, why this residue is so special? Please explain. For other residues, the increase was between 3%-10% (except for D86), did this minor difference has statistical significance?***

This is a good point. We have added the level of statistical significance for the increase in calcium ion coordination for residues E416 and D77 as these are statistically significant and have made it clear that more data is needed to ascertain with confidence the changes at the other acidic residues. It is unclear why D77 is particularly susceptible to higher cation coordination in the presence of CDCA. However, we do note that the phenomenon of cation coordination to acidic residues within the channel is striking and reproducible. We note that D77 is not well-conserved (while E416 is) and thus the higher levels of coordination to these specific residues seen here is likely a stochastic effect.

***The authors presented the orientation of the ligands in the channel by showing these ligands from different frames in one figure. However, I am afraid this might not be a perfect way to describe the ligand orientations. I suggest the authors to define other ways, such as define vectors and angles/dihedrals or show densities of specific atoms.***

An attempt was made to use the orientation of the vector defined by the longest axis of each molecule as a function of z to define the orientation of the ligands as they translocate. However, this information did not prove to be useful; for CDCA the important interaction is between the carboxylate moiety and the protein-bound calcium ion. CDCA rotates about this carboxylate-calcium coordination site as it translocates, resulting in a wide range of relative orientations of the long axis of the molecule, even in regions where the CDCA is coordinated to a bound calcium. We therefore generated occupancy maps for specific atoms within each of the substrate molecules (a hydroxyl oxygen in erythromycin and a carboxylate oxygen in CDCA) across the steered MD simulations. These new figures can be found in the supplementary information (new supplementary figures S12 and S15). For CDCA, the occupancy map of the carboxylate atom displays localised regions of high occupancy proximal to acidic residues. For erythromycin, the occupancy of the selected hydroxyl oxygen is delocalised and more uniformly distributed through the channel.

***Please clarify the concentration of calcium ions used in the work. The concentration of KCL was mentioned but not for the calcium ions.***

The concentration of calcium ions in the system is 125 mM. This concentration has been included in the methods section (first paragraph in the subsection titled ***“System Construction”***)

## Reviewer 2

*This manuscript nicely describes the influence of structure and function for CmeC by lipid membrane models, ions, efflux of drugs. There is an extensive set of simulations that albeit not fully equilibrated provide some details on structural influences on CmeC by various conditions. Although I find this manuscript publishable, my comments and suggestions below should be addressed.*

***Outer membrane (OM) mimic: It is nice to see that the simulations utilize a mimic for the outer membrane with LOS, which better represents the natural environment for CmeC. However, the membrane model used is a mimic and not representative of the natural OM of C. jejuni. The authors should make this clear in the main document.***

We thank the reviewer for this key point. This limitation was stated later in the document (in the section titled ***“Limitations”***). A clarifying statement on the lack of lysophospholipids in our model has been added in the first paragraph of the subsection titled ***“CmeC Stability within the Membrane”*** so that this is now addressed earlier in the manuscript.

***Equilibration (lack of): The authors are very clear and open about their research and that the protein is still changing in structure. It would be nice to provide a bit more detail on what regions of the protein are still changing. Moreover, does this relate to the crystal structure conditions vs. the more natural conditions simulated in this work? Some more physical insight into the inability to stabilize the structure would be good.***

The continued rise in RMSD is primarily driven by the β-barrel. This is the domain in which the crystallisation and simulation environments are most different; in the crystal structure, the β-barrel is surrounded by detergent molecules [(20)](https://www.zotero.org/google-docs/?sjICuV), but in the simulation environment the β-barrel is embedded in an asymmetric bilayer of phospholipids and LOS molecules. This is in contrast to the periplasmic domains (α-barrel and coiled-coil domains) which are soluble and therefore surrounded by water in both environments. In addition to this, the model membrane environment is itself slow to equilibrate due to the slow lateral motion and extensive cation-mediated cross-linking of LOS molecules. Both of these factors lead to a slow equilibration of the β-barrel domain of CmeC. A statement has been added to the second paragraph of the subsection titled ***“CmeC Stability within the Membrane”*** to reflect this.

## Reviewer 3

*The manuscript entitled 'Conformational dynamics and putative substrate extrusion pathways of the N- glycosylated outer membrane factor CmeC from Campylobacter jejuni' by authors Kahlan Newman and Syma Khalid is an interesting study of the dynamics of a previously not dynamically investigated RND efflux pump outer duct. The work is technically sound in general, very well written, and provides interesting insights on the dynamics of loops, N-glycans and the trimer as a whole. Of particular interest is the role of the inner pore lining, which is especially rich in acidic side chains. The authors ought to be commended for using realistic outer membrane models and post-translational modification. I only have a few questions:*

***The default model for calcium ion is known to overestimate protein-ion affinity (see for example doi: 10.1038/s41467-020-14573-w). The density and position of calcium ions near the protein is quite important for the results of this work - can the authors please comment why they chose the default model and discuss any possible implications for the results?***

We thank the reviewer for raising this important point. Limitations of the force field with respect to multivalent cations were also raised as a concern by Reviewer 1. It is possible that the coordination of calcium has been overestimated here; systematic overestimation of binding free energies between proteins and divalent cations has been documented [(4)](https://www.zotero.org/google-docs/?F6k7q5). However, updates to the Charmm36 force field have addressed issues with the non-bonded interactions between cations and carboxylate moieties by increasing the Lennard-Jones parameter, σ [(5)](https://www.zotero.org/google-docs/?SNWk5j). Similar NBFIX force field corrections have been shown to drastically improve estimations of the cation-protein binding free energy [(6)](https://www.zotero.org/google-docs/?9Gwxw8). We have used the CHARMM36m force field with these updated non-bonded parameters, and to the best of our knowledge this model is appropriate for the scope of this paper. We note that in the paper referred to by the reviewer, there were no calcium ion permeation events as the calcium was bound too tightly within the protein [(4)](https://www.zotero.org/google-docs/?zSZXhc). This was not the case in our simulations, as calcium ions were observed to bind and unbind within CmeC. To address the reviewers’ concerns, we have added an extra paragraph on our use of this model in the subsection titled ***“Cation binding sites within the channel”****.* Should we wish to calculate binding free energies or quantify conductance through the channel, a polarisable [(7,8)](https://www.zotero.org/google-docs/?p5PU7d) or multipoint [(4)](https://www.zotero.org/google-docs/?k5yxJG) model would be used to ensure reasonable accuracy.

***I wonder why no attempt was made to estimate the free energy surface for substrate translocation by using the steered MD results along with the Jarzynski equality rather than by umbrella sampling since the steered MD trajectories already exist. Was there poor convergence for instance?***

While we could have used the Jarzynski equality to estimate the free energy surface, we decided against its use. First and foremost, “to obtain the observable effects the systems have to be small, since otherwise the fluctuations of the work values become too small to observe” [(21)](https://www.zotero.org/google-docs/?0ydYzw); estimation of the energy surface via the Jarzynski equality becomes unreliable for large systems, and the system used here contains close to 1 million atoms. Further to this, while we do have the steered molecular dynamics trajectories, we only have 9 for each substrate. For such a long and wide channel we do not feel confident that our sampling is sufficient to estimate the energy surface reliably using the Jarzynski equality. Simulation lengths likely need to be around the same, if not an order of magnitude greater, than those used in umbrella sampling in order to extract an accurate energy surface [(22)](https://www.zotero.org/google-docs/?UlxINY). To ensure convergence, the free energy should be calculated from (and be consistent across) multiple pulling rates [(23)](https://www.zotero.org/google-docs/?J7QsCW), but we have only sampled a single pulling rate (0.1 nm ns^-1^). In addition, long-range electrostatic interactions can cause errors in the estimation of the free energy surface [(22)](https://www.zotero.org/google-docs/?a8DD0P). Electrostatic interactions play a key role in the interactions between CmeC and CDCA, and as such it would not be appropriate to use a method that is heavily affected by them. Nevertheless this is an important point and we have now added a sentence to the ***Limitations*** section of the manuscript to address this.

***Ion attraction sites, the electrostatic properties of the gating region, and pore gating in a similar outward efflux duct from Neisseria gonorrhoeae were studied by MD in doi: 10.1038/s41598-017-16995-x ; I think this paper is relevant previous work and the authors may want to discuss it.***

The reviewer is correct that this elegant paper is relevant; it was an oversight on the authors’ parts that this was not included. The paper referenced here identifies that MtrE, despite its open conformation in the x-ray crystal structure, behaves similarly to TolC and OprM when simulated under equilibrium conditions. The simulated MtrE adopts the closed state when unbound from the rest of the efflux machinery, and is gated at the periplasmic entrance.However, unlike TolC and OprM, MtrE displays only a slight preference for cations, and is distinct in that it does not have extensive positively or negatively charged patches within the channel. We have referenced this paper in the introduction alongside simulation studies of TolC and OprM.

***Are any inhibitors of this outward duct known and if so, where do they bind?***

We are not aware of any inhibitors of this specific outer membrane factor from *Campylobacter jejuni*. However, there are known orthosteric inhibitors of the homologue TolC. Multivalent cationic species are known to be coordinated by the conserved rings of acidic residue at the periplasmic entrance. For example, Higgins *et al.* showed that [Co(NH_3_)_6_]^3+^ acts as an inhibitor for TolC. This complex is coordinated by the two rings of aspartate residues in TolC, preventing the opening of the coiled-coil region; it is shown that binding of this complex blocks the channel, reducing conductance [(1)](https://www.zotero.org/google-docs/?1iQe9g). Additionally, Masi *et al.* have shown that the pore-forming activity of TolC (from *Enterobacter aerogenes*) is reduced by divalent cations entering the channel from the extracellular side [(2)](https://www.zotero.org/google-docs/?a6syh6). We have added an additional sentence to the first paragraph of the subsection titled ***“Cation binding sites within the channel”*** to include references to the above studies. It is also of note that colicins have recently been shown to ‘plug’ TolC in *E. coli* to inhibit efflux [(24)](https://www.zotero.org/google-docs/?lhWNHd); analogous bacteriocin-outer membrane factor interactions have not been described in *C. jejuni*, though it is possible that such inhibitors exist.

**References**

[1.	Higgins MK, Eswaran J, Edwards P, Schertler GFX, Hughes C, Koronakis V. Structure of the Ligand-blocked Periplasmic Entrance of the Bacterial Multidrug Efflux Protein TolC. J Mol Biol. 2004 Sep 17;342(3):697–702.](https://www.zotero.org/google-docs/?U6svnI)

[2.	Masi M, Saint N, Molle G, Pagès JM. The Enterobacter aerogenes outer membrane efflux proteins TolC and EefC have different channel properties. Biochim Biophys Acta BBA - Biomembr. 2007 Oct 1;1768(10):2559–67.](https://www.zotero.org/google-docs/?U6svnI)

[3.	Andersen C, Koronakis E, Hughes C, Koronakis V. An aspartate ring at the TolC tunnel entrance determines ion selectivity and presents a target for blocking by large cations. Mol Microbiol. 2002;44(5):1131–9.](https://www.zotero.org/google-docs/?U6svnI)

[4.	Zhang A, Yu H, Liu C, Song C. The Ca2+ permeation mechanism of the ryanodine receptor revealed by a multi-site ion model. Nat Commun. 2020 Feb 17;11(1):922.](https://www.zotero.org/google-docs/?U6svnI)

[5.	Newest CHARMM36 port for GROMACS - Third party tools and files [Internet]. GROMACS forums. 2020 [cited 2022 Oct 20]. Available from: https://gromacs.bioexcel.eu/t/newest-charmm36-port-for-gromacs/868](https://www.zotero.org/google-docs/?U6svnI)

[6.	Liu H, Fu H, Chipot C, Shao X, Cai W. Accuracy of Alternate Nonpolarizable Force Fields for the Determination of Protein–Ligand Binding Affinities Dominated by Cation−π Interactions. J Chem Theory Comput. 2021 Jul 13;17(7):3908–15.](https://www.zotero.org/google-docs/?U6svnI)

[7.	Jing Z, Liu C, Qi R, Ren P. Many-body effect determines the selectivity for Ca2+ and Mg2+ in proteins. Proc Natl Acad Sci. 2018 Aug 7;115(32):E7495–501.](https://www.zotero.org/google-docs/?U6svnI)

[8.	Jing Z, Qi R, Liu C, Ren P. Study of interactions between metal ions and protein model compounds by energy decomposition analyses and the AMOEBA force field. J Chem Phys. 2017 Oct 28;147(16):161733.](https://www.zotero.org/google-docs/?U6svnI)

[9.	Kastowsky M, Sabisch A, Gutberlet T, Bradaczek H. Molecular modelling of bacterial deep rough mutant lipopolysaccharide of Escherichia coli. Eur J Biochem. 1991;197(3):707–16.](https://www.zotero.org/google-docs/?U6svnI)

[10.	Clifton LA, Skoda MWA, Le Brun AP, Ciesielski F, Kuzmenko I, Holt SA, et al. Effect of Divalent Cation Removal on the Structure of Gram-Negative Bacterial Outer Membrane Models. Langmuir. 2015 Jan 13;31(1):404–12.](https://www.zotero.org/google-docs/?U6svnI)

[11.	Raetz CRH, Reynolds CM, Trent MS, Bishop RE. Lipid A Modification Systems in Gram-Negative Bacteria. Annu Rev Biochem. 2007;76(1):295–329.](https://www.zotero.org/google-docs/?U6svnI)

[12.	Oliveira RG, Schneck E, Quinn BE, Konovalov OV, Brandenburg K, Gutsmann T, et al. Crucial roles of charged saccharide moieties in survival of gram negative bacteria against protamine revealed by combination of grazing incidence x-ray structural characterizations and Monte Carlo simulations. Phys Rev E. 2010 Apr 2;81(4):041901.](https://www.zotero.org/google-docs/?U6svnI)

[13.	Murata T, Tseng W, Guina T, Miller SI, Nikaido H. PhoPQ-Mediated Regulation Produces a More Robust Permeability Barrier in the Outer Membrane of Salmonella enterica Serovar Typhimurium. J Bacteriol. 2007 Oct 15;189(20):7213–22.](https://www.zotero.org/google-docs/?U6svnI)

[14.	Jefferies D, Hsu PC, Khalid S. Through the Lipopolysaccharide Glass: A Potent Antimicrobial Peptide Induces Phase Changes in Membranes. Biochemistry. 2017 Mar 21;56(11):1672–9.](https://www.zotero.org/google-docs/?U6svnI)

[15.	Nascimento A, Pontes FJS, Lins RD, Soares TA. Hydration, ionic valence and cross-linking propensities of cations determine the stability of lipopolysaccharide (LPS) membranes. Chem Commun. 2013 Nov 28;50(2):231–3.](https://www.zotero.org/google-docs/?U6svnI)

[16.	Pontes FJS, Rusu VH, Soares TA, Lins RD. The Effect of Temperature, Cations, and Number of Acyl Chains on the Lamellar to Non-Lamellar Transition in Lipid-A Membranes: A Microscopic View. J Chem Theory Comput. 2012 Oct 9;8(10):3830–8.](https://www.zotero.org/google-docs/?U6svnI)

[17.	Santos DES, Pol-Fachin L, Lins RD, Soares TA. Polymyxin Binding to the Bacterial Outer Membrane Reveals Cation Displacement and Increasing Membrane Curvature in Susceptible but Not in Resistant Lipopolysaccharide Chemotypes. J Chem Inf Model. 2017 Sep 25;57(9):2181–93.](https://www.zotero.org/google-docs/?U6svnI)

[18.	Kesireddy A, Pothula KR, Lee J, Patel DS, Pathania M, van den Berg B, et al. Modeling of Specific Lipopolysaccharide Binding Sites on a Gram-Negative Porin. J Phys Chem B. 2019 Jul 11;123(27):5700–8.](https://www.zotero.org/google-docs/?U6svnI)

[19.	Clifton LA, Holt SA, Hughes AV, Daulton EL, Arunmanee W, Heinrich F, et al. An Accurate In Vitro Model of the E. coli Envelope. Angew Chem Int Ed. 2015;54(41):11952–5.](https://www.zotero.org/google-docs/?U6svnI)

[20.	Su CC, Radhakrishnan A, Kumar N, Long F, Bolla JR, Lei HT, et al. Crystal structure of the Campylobacter jejuni CmeC outer membrane channel. Protein Sci. 2014;23(7):954–61.](https://www.zotero.org/google-docs/?U6svnI)

[21.	Cohen EGD, Mauzerall D. A note on the Jarzynski equality. J Stat Mech Theory Exp. 2004 Jul;2004(07):P07006.](https://www.zotero.org/google-docs/?U6svnI)

[22.	Baştuğ T, Kuyucak S. Application of Jarzynski’s equality in simple versus complex systems. Chem Phys Lett. 2007 Mar 9;436(4):383–7.](https://www.zotero.org/google-docs/?U6svnI)

[23.	Kuang Z, Singh KM, Oliver DJ, Dennis PB, Perry CC, Naik RR. Gamma estimator of Jarzynski equality for recovering binding energies from noisy dynamic data sets. Nat Commun. 2020 Nov 2;11(1):5517.](https://www.zotero.org/google-docs/?U6svnI)

[24.	Budiardjo SJ, Stevens JJ, Calkins AL, Ikujuni AP, Wimalasena VK, Firlar E, et al. Colicin E1 opens its hinge to plug TolC. Drew D, Faraldo-Gómez JD, Drew D, van den Berg B, Jakes K, editors. eLife. 2022 Feb 24;11:e73297.](https://www.zotero.org/google-docs/?U6svnI)
